# Hypertension and grading are important risk factors for occult blood loss in hip arthroplasty: a retrospective study analysis

**DOI:** 10.3389/fsurg.2025.1694480

**Published:** 2026-01-30

**Authors:** Erhui Song, Feng Gao, Guanghe Zhang

**Affiliations:** Department of Orthopedics, Taizhou Fourth People’s Hospital, Clinical College of Yangzhou University Medical College, Taizhou, Jiangsu, China

**Keywords:** hypertension, occult blood loss, retrospective study, total hip arthroplasty, transfusion risk

## Abstract

**Objective:**

To quantify the impact of hypertension and its grading on occult blood loss (HBL) during total hip arthroplasty (THA) and to offer clinical guidance for minimizing HBL.

**Methods:**

Baseline data from femoral neck fracture patients treated with THA between January 2018 and December 2022 were included. SPSS 26.0 statistical software was used for correlation analysis employing statistical methods, including independent samples *t*-test, Pearson correlation, and multiple linear regression, to identify risk factors for elevated postoperative HBL in THA patients. Hypertension severity was categorized according to international guidelines to investigate the effect of hypertension grading on HBL.

**Results:**

The mean perioperative bleeding (TBL) among all patients was 1,123.39 ± 518.89 mL, and the mean HBL was 923.93 ± 489.04 mL, which accounted for 78.76% ± 16.09% of the TBL. HBL was significantly higher in hypertensive patients (957.98 ± 509.72 mL vs. 895.94 ± 469.97 mL, *P* = 0.042). Multiple linear regression analysis revealed that hypertension was an independent predictor of HBL (*P* = 0.030). Grade 2 hypertension increased HBL by 11.2% (996.46 ± 573.80 mL, *P* = 0.046), while grade 3 hypertension further increased HBL by 18.7% (1,063.76 ± 584.11 mL, *P* = 0.044). Hypoalbuminemia had a clinically relevant, but not statistically significant, synergistic effect with hypertension (*Δ*HBL = 119.60 mL, *P* = 0.297).

**Conclusion:**

Hypertension ≥ grade 2 (systolic blood pressure ≥ 160 mmHg) independently exacerbates HBL in THA patients through a dose-response relationship. It is recommended that preoperative systolic blood pressure be maintained below 160 mmHg, and metabolic status be optimized to reduce the risk of blood transfusion.

## Background

As total hip arthroplasty (THA) continues to mature and gain widespread adoption, it has become the gold-standard intervention for patients with end-stage hip diseases, including femoral head necrosis and femoral neck fractures (1). THA provides significant pain relief and effectively restores motor function by enhancing the biomechanical function of the hip joint (2). However, as clinical practice deepens and perioperative management improves, previously overlooked postoperative complications have gradually emerged. Among these, hidden blood loss (HBL) remains a critical yet under-recognized issue.

The concept of HBL was first quantitatively defined by Sehat et al. as the “calculated hemoglobin mass loss exceeding visible surgical blood loss and postoperative drainage” (3). Current evidence indicates that HBL accounts for 30%–50% of total blood loss during joint replacement surgery, primarily due to extravasation into tissue spaces, accumulation within the residual joint, and hemolysis (4). High levels of HBL lead to symptomatic anemia requiring transfusion, prolonged hospitalization, increased costs, and an elevated risk of surgical site infection and venous thromboembolism (5–9).

Hypertension, a modifiable risk factor, contributes to occult blood loss through several pathophysiological mechanisms. Available research evidence indicates that hypertension exacerbates HBL through endothelial dysfunction, which impairs vasoconstriction (10), an altered coagulation-fibrinolytic balance (11), and microvascular fragility in chronic hypertension (12). A recent retrospective study of 206 THA patients demonstrated that hypertension was a risk factor for postoperative blood loss, with a column-line graph weight of 23 (13). Despite these associations, few studies have directly compared HBL patterns between hypertensive and non-hypertensive cohorts, or conducted longitudinal studies comparing different blood pressure classifications and HBL. However, some studies have suggested that hypertension is not an independent risk factor for HBL (14, 15). More crucially, existing evidence predominantly treats hypertension as a binary variable, with few studies investigating the potential graded relationship between its severity and the volume of HBL. Therefore, investigating the impact of hypertension grading on HBL is critically important.

The aim of this study was to quantify differences in HBL between hypertensive and non-hypertensive THA patients, explore the relationship between different degrees of hypertension and HBL, and propose strategies for blood pressure management in THA patients to improve surgical prognostic care and reduce the need for blood transfusions.

## Methods

### Patients

A total of 952 patients who underwent hip replacement between January 2018 and June 2022 at the People's Hospital of North Jiangsu Province and the Fourth People's Hospital of Taizhou City were included. All patients were managed before and after surgery by orthopedic surgeons from the People's Hospital of North Jiangsu and the Fourth People's Hospital of Taizhou City. This retrospective study was conducted in accordance with the Declaration of Helsinki and was approved with a waiver of informed consent by the Ethics Committees/Institutional Review Boards of Yangzhou University Subei People's Hospital and the Fourth People's Hospital of Taizhou City (Approval/Waiver Nos. 2025ky090 and 2023-EC/TZFH-048).

The inclusion criteria were as follows: (1) Patients with a confirmed diagnosis of femoral neck fracture requiring THA, as determined by imaging and clinical evaluation; (2) Patients who had not previously undergone hip surgery; (3) Age ≥ 18 years; 4. No serious cardiovascular or cerebrovascular issues within 6 months prior to surgery; and 5. Possession of a complete and accurate record of information.

The exclusion criteria were: (1) Hematologic and coagulation disorders; (2) Patients who received a blood transfusion within 4 weeks prior to surgery; (3) Patients with a history of or current oncologic diseases; (4) Presence of other contraindications to surgery; (5) Withdrawal during the procedure or incomplete data.

### Data collection

Data were collected from all patients, including gender, age, body mass index (BMI), operative time hypertension, diabetes, cardiovascular diseases, preoperative red blood cells (RBCs), preoperative hemoglobin (HB), preoperative hematocrit (HCT), preoperative platelets (PLTs), preoperative plasminogen time (PT), and preoperative activated partial thromboplastin time (APTT), preoperative international normalized ratio (INR), preoperative thrombin time (TT), preoperative fibrinogen (FIB), preoperative calcium, preoperative C-reactive protein (CRP), preoperative Erythrocyte Sedimentation Rate (ESR), preoperative DD polymers, preoperative albumin (ALB), American Society of Anesthesiologists Score (ASA) rating, intraoperative blood loss, operative time, 3-day postoperative HB and 3-day postoperative HCT. day HCT, etc. Hypertensive patients were controlled with preoperative medication to <140/90 mmHg.

To ensure the objectivity and accuracy of data collection, data were independently extracted from the hospital medical record system by two specialized surgeons. In cases of disputes over data processing, the two physicians reached a preliminary consensus and then discussed with a third physician until a collective agreement was reached.

### Blood transfusion management

The hospital adheres to a restrictive blood transfusion management protocol in accordance with the Clinical Blood Transfusion Specifications (16). When the patient's hemoglobin (Hb) level is less than 70 g/L, or when the hemoglobin level is less than 80 g/L with severe anemia symptoms (such as extreme weakness, chest pain, extreme pallor, or hemorrhage), or in the presence of hemodynamic instability (such as a heart rate exceeding 100 beats per minute or systolic blood pressure below 90 mmHg), a blood transfusion will be administered. In this study, all patiens received leukocyte-reduced red blood cell transfusions.

#### Blood pressure management

This study implemented a standardized preoperative blood pressure management protocol for all enrolled patients with hypertension. In accordance with Chinese clinical guidelines, the preoperative blood pressure control targets were set as follows: <160/100 mmHg for general adult patients, and <150/90 mmHg for patients aged ≥60 years. To ensure these targets were met, the antihypertensive regimen was initiated and optimized at least two weeks prior to surgery during outpatient visits, accompanied by daily blood pressure monitoring. On the morning of the surgery, hypertensive patients were instructed to take their usual antihypertensive medications with a small sip of water to maintain hemodynamic stability and ensure safer surgical conditions.

### Calculation of blood volume

The clinical estimation of the patient's blood volume (EBV) is performed using the formula EBV = K₁ × height³ (m) + K₂ × weight (kg) + K₃, with gender-specific coefficients: K₁ = 0.3669, K₂ = 0.03219, K₃ = 0.6041 for males, and K₁ = 0.3561, K₂ = 0.03308, K₃ = 0.1833 for females (17). After calculating the EBV, total blood loss (TBL) was determined using the Gross formula: TBL = EBV × (preoperative HCT—postoperative HCT)/HCT mean (18). Hidden blood loss (HBL) was then calculated using the formula HBL = TBL + blood transfusion—visible bleeding (intraoperative bleeding + postoperative drainage). Intraoperative blood loss was measured as the sum of the blood collected in the suction bottle and the blood absorbed by the surgical gauze.

### Statistical methods

All statistical analyses were conducted using SPSS (version 26.0, IBM, USA). For data presentation, continuous variables were expressed as mean ± standard deviation (i.e., M ± SD), while categorical variables were presented as counts and percentages [*N* (%)]. The independent samples *t*-test was used to calculate significant differences in HBL between groups, while Pearson's correlation coefficient was used to analyze differences between measurements. A one-way analysis of variance (ANOVA) was employed for comparisons between groups. Statistically significant variables were selected for multiple linear regression analysis to identify independent risk factors associated with HBL. The results were considered statistically significant when the *p*-value was less than 0.05, in accordance with the statistical criteria set for this study.

## Results

### Perioperative parameters

Data from 1,035 hypertensive and non-hypertensive patients were retrospectively analyzed, including 364 (35.2%) males and 671 (64.8%) females. Of these, 467 (45.1%) were hypertensive patients with a mean age of 62.89 ± 11.36 years and a mean BMI of 23.25 ± 3.43, while 568 (54.9%) were non-hypertensive patients with a mean age of 69.51 ± 8.40 years and a mean BMI of 24.68 ± 3.33. The hypertensive occult blood loss was 957.98 ± 509.72 mL, while the non-hypertensive occult blood loss was 895.94 ± 469.97 mL, with further details provided in Table 1. Table 2 presents data on TBL, visible blood loss, HBL, and the percentage of HBL calculated for HGB loss, HCT loss, and the number of patients with preoperative and postoperative HGB <100 g/L. The mean TBL was 1,123.39 ± 518.89 mL, and the mean HBL was 923.93 ± 489.04 mL, which accounted for 78.76% ± 16.09% of the TBL. Thirty-six patients (3.5%) had preoperative Hb <100 g/L, which increased to 268 patients (25.9%) after surgery.

**Table 1:** Information on baseline characteristics of patients with hypertension and non-hypertension.

Factors	Groups	Non-hypertension (*n* = 568)	Hypertension (*n* = 467)	t/x2	*P*
Age		62.89 ± 11.36	69.51 ± 8.40	−10.764	<0.001
BMI		23.25 ± 3.43	24.68 ± 3.33	−6.754	<0.001
Operative time		76.89 ± 27.88	75.19 ± 27.29	0.990	0.322
RBC		4.26 ± 0.58	4.28 ± 0.57	−0.636	0.525
PLT		196.64 ± 72.23	197.33 ± 66.90	−0.158	0.875
HB		129.81 ± 17.30	130.50 ± 17.01	−0.646	0.519
HCT		38.83 ± 4.78	39.07 ± 5.98	−0.698	0.485
PT		13.05 ± 0.87	13.11 ± 1.43	−0.759	0.488
PTINR		1.07 ± 1.57	1.27 ± 3.32	−1.193	0.234
APTT		38.18 ± 5.11	37.20 ± 5.28	3.003	0.003
TT		17.47 ± 1.64	17.39 ± 1.53	0.792	0.429
FIB		3.71 ± 0.94	3.79 ± 1.08	−1.270	0.204
DD polymer		3.04 ± 4.49	3.02 ± 4.19	0.065	0.948
Calcium		2.30 ± 0.12	2.35 ± 0.69	−1.717	0.086
CRP		14.50 ± 23.72	16.56 ± 23.03	−1.407	0.160
ESR		22.67 ± 20.72	21.81 ± 18.31	0.704	0.482
ALB		43.33 ± 4.79	43.21 ± 4.65	0.411	0.681
Drainage volume		11.86 ± 74.56	16.02 ± 75.59	−0.888	0.375
Blood transfusion volume		102.29 ± 236.25	102.57 ± 243.618	−0.019	0.985
Intraoperative blood loss		191.17 ± 135.17	181.37 ± 9.35	1.320	0.187
Visible blood loss		203.03 ± 165.23	197.39 ± 136.36	0.591	0.555
EBV		3.92 ± 0.64	4.06 ± 0.65	−3.399	0.001
Hospitalization days		9.36 ± 3.49	10.05 ± 4.35	−2.777	0.006
Gender	Female	369 (65.0%)	302 (64.7%)	0.010	0.921
	Male	199 (35.0%)	165 (35.3%)		
Side of surgery	Left	301 (53.0%)	237 (50.7%)	0.517	0.472
	Right	267 (47.0%)	230 (49.3%)		
Diabetes	Without	520 (91.5%)	366 (78.4%)	36.108	<0.001
	With	48 (8.5%)	101 (21.6%)		
Cardiovascular diseases	Without	506 (89.1%)	356 (76.2%)	30.414	<0.001
	With	62 (10.9%)	111 (23.8%)		
Smoking history	Without	538 (94.7%)	442 (94.6%)	0.003	0.959
	With	30 (5.3%)	25 (5.4%)		
Drinking history	Without	545 (96.0%)	447 (95.7%)	0.035	0.851
	With	23 (4.0%)	20 (4.3%)		
ASA	<3	460 (81.0%)	286 (61.2%)	49.641	<0.001
	≥3	108 (19.0%)	181 (38.8%)		
Osteoporosis	Without	387 (68.1%)	300 (64.2%)	1.741	0.187
	With	181 (31.9%)	167 (35.8%)		
Drain tube	Without	546 (96.1%)	438 (93.8%)	2.987	0.084
	With	22 (3.9%)	29 (6.2%)		
Albumin supplementation	Without	543 (95.6%)	452 (96.8%)	0.976	0.323
	With	25 (4.4%)	15 (3.2%)		

**Table 2:** Parameters related to perioperative blood loss.

Parameter	Mean ± SD or *N* (%)
Hemoglobin loss (g/L)	30.86 ± 12.80
Hematocrit level loss (%)	8.86 ± 4.61
Hemoglobin <100 g/L preoperatively (*n*)	36 (3.5%))
Hemoglobin <100 g/L postoperatively (*n*)	268 (25.9%)
Calculated total blood loss (mL)	1,123.39 ± 518.89
Visible blood loss (mL)	200.49 ± 152.83
Hidden blood loss (mL)	923.93 ± 489.04
Percentage of hidden blood loss in total (%)	78.76 ± 16.09

### Univariate analysis

According to the results of univariate analysis, HBL was statistically significant (*P* < 0.05) with respect to age, gender, BMI, hypertension, preoperative RBC, preoperative HB, preoperative HCT, preoperative FIB, preoperative DD, preoperative CA, preoperative CRP, preoperative ALB, operative time, ASA, and EBV. There was no statistically significant difference with respect to preoperative PLT, preoperative PT, preoperative PTINR, preoperative APTT, preoperative TT, preoperative ESR, side of surgery, diabetes, cardiovascular diseases, smoking history, drinking history, osteoporosis, drainage volume, intraoperative blood loss, and hospitalization days. The measured data and calculated results are presented in Tables 3, 4, respectively.

**Table 3:** Results of HBL and Pearson's correlation analysis for different count data sets.

Parameter	Mean ± SD or *N* (%)	Pearson value	*P* value
Age	65.88 ± 10.65	−0.111	<0.001
BMI	23.89 ± 3.46	0.064	0.039
Operative time	76.12 ± 27.61	0.123	<0.001
RBC	4.27 ± 0.58	0.168	<0.001
PLT	196.95 ± 69.84	−0.027	0.380
PT	13.08 ± 1.16	−0.048	0.119
PTINR	1.16 ± 2.51	0.032	0.304
APTT	37.74 ± 5.21	−0.043	0.163
TT	17.44 ± 1.59	−0.035	0.260
FIB	3.74 ± 1.01	−0.075	0.016
DD polymer	3.03 ± 4.36	−0.129	<0.001
Calcium	2.31 ± 0.13	0.196	<0.001
CRP	15.43 ± 23.42	−0.128	<0.001
ESR	22.28 ± 19.66	−0.017	0.581
HB	130.12 ± 17.16	0.185	<0.001
HCT	38.94 ± 5.35	0.192	<0.001
ALB	43.28 ± 4.72	0.192	<0.001
Drainage volume	13.74 ± 75.02	−0.034	0.278
Intraoperative blood loss	186.75 ± 118.91	0.059	0.057
Visible blood loss	200.49 ± 152.83	0.029	0.343
EBV	3.98 ± 0.65	0.194	<0.001
Hospitalization days	9.67 ± 3.92	0.043	0.164

**Table 4:** Correlation analysis results of HBL and independent samples *t*-test for different count data groups in the perioperative period.

Index	Groups	*N*	HBL(mL)	T	*P*
Gender	Female	671	865.99 ± 475.79	−5.240	<0.001
	Male	364	1,030.73 ± 495.76		
Side of surgery	Left	538	931.61 ± 484.21	0.526	0.599
	Right	497	915.61 ± 494.58		
Hypertension	Without	568	895.94 ± 469.97	−2.034	0.042
	With	467	957.98 ± 509.72		
Diabetes	Without	886	934.35 ± 479.37	1.673	0.095
	With	149	861.98 ± 540.61		
Cardiovascular diseases	Without	862	915.06 ± 473.21	−1.165	0.245
	With	173	968.14 ± 560.74		
Smoking history	Without	980	917.85 ± 490.48	−1.691	0.091
	With	55	1,032.35 ± 453.16		
Drinking history	Without	992	919.10 ± 490.99	−1.526	0.127
	With	43	1,035.30 ± 431.90		
ASA	<3	746	960.47 ± 472.72	−3.888	<0.001
	≥3	289	829.61 ± 517.88		
Osteoporosis	Without	687	922.50 ± 483.42	0.132	0.895
	With	348	926.75 ± 500.64		
Drain tube	Without	984	926.08 ± 493.63	0.620	0.535
	With	51	882.53 ± 391.85		
Albumin supplementation	Without	995	920.06 ± 490.82	−1.270	0.204
	With	40	1,020.18 ± 436.93		

### Multiple linear regression analysis

Postoperative HBL was used as the dependent variable, with the 15 statistically significant variables mentioned above serving as independent variables in the multiple linear regression analysis. The results indicated that HBL was associated with BMI, hypertension, operative time, preoperative calcium, preoperative HCT, preoperative ALB, and preoperative EBV (Table 5).

**Table 5:** Results of multiple linear regression analysis.

Index	Coefficient B	Beta (*β*)	T Value	*P* value
Gender	−27.235	−0.027	−0.510	0.610
Age	0.716	0.016	0.407	0.684
BMI	−13.521	−0.096	−2.359	0.019
Hypertension	70.239	0.072	2.174	0.030
Operative time	1.647	0.093	3.067	0.002
RBC	−32.969	−0.039	−0.627	0.531
HB	−2.449	−0.086	−1.186	0.236
HCT	14.603	0.160	2.827	0.005
FIB	−19.463	−0.040	−1.149	0.251
DD polymer	−4.475	−0.040	−1.167	0.244
Calcium	432.194	0.111	3.061	0.002
CRP	−0.571	−0.027	−0.750	0.453
ALB	8.376	0.081	2.109	0.035
ASA	−48.082	−0.053	−1.528	0.127
EBV	148.307	0.196	3.329	0.001

### Impact of hypertension stratification

To further investigate the impact of hypertension severity on HBL, hypertension was classified into 3 grades according to international standards (19). Stratification analysis showed that the HBL of grade 1 hypertensive patients (*n* = 319) was 932.74 ± 475.29 mL, which was not significantly different from that of the non-hypertensive group (895.94 ± 469.97 mL, *P* = 0.265). HBL in grade 2 hypertensive patients (*n* = 113) significantly increased to 996.46 ± 573.80 mL, reflecting an 11.2% increase compared to the non-hypertensive group (*P* = 0.046). Blood loss in grade 3 hypertensive patients (*n* = 35) further increased to 1,063.76 ± 584.11 mL, corresponding to an 18.7% increase (*P* = 0.044) (Table 6). In addition, among patients with combined hypoalbuminemia, HBL increased by 119.60 mL compared with the non-hypoalbuminemia group (a relative increase of 11.3%), but this difference was not statistically significant (*P* = 0.297) (Table 7).

**Table 6:** Exploring the effect of severity of hypertension.

Subgroup	Groups	*N*	HBL (mL)	Rate of increase	Intergroup *p*-value	Comparison with non-hypertension group *p* value
Non-hypertension		568	895.94 ± 469.97			
Hypertension					0.062	
	1	319	932.74 ± 475.29	+4.1%		0.265
	2	113	996.46 ± 573.80	+11.2%		0.046
	3	35	1,063.76 ± 584.11	+18.7%		0.044

**Table 7:** Exploring the effect of hypertension combined with hypoalbuminemia on HBL.

Group	*n*	HBL (mL)	Mean spread and increase	*P*
Non-hypertension	1,015	921.62 ± 488.92 mL		0.297
Combined hypertension	20	1,041.22 ± 493.60 mL	119.60 mL (11.30%)	

## Discussion

With ongoing advances in medical technology and socio-economic development, there has been a growing emphasis on perioperative management and the postoperative quality of life for patients. In particular, addressing complications such as occult blood loss (HBL) and the need for blood transfusions following total hip arthroplasty (THA) has become a key area of focus (20). In the field of THA, previous literature has generally reported that hidden blood loss (HBL) can account for 50% to more than 80% of total blood loss (TBL) (4, 21), underscoring its critical role in perioperative blood loss. While significant progress has been made in developing strategies to minimize overt blood loss in THA patients (22) the role of HBL, particularly the impact of hypertension and its stratification in relation to HBL, remains inadequately understood. To address this gap, this study analyzes clinical data from 1,035 patients using the Gross equation, a widely used method in orthopedic research. Compared to previous studies, a more precise risk-assessment model for THA blood loss was developed by excluding patients with coagulation disorders, implementing stringent blood pressure control, and monitoring a range of physiological parameters (20, 23). The results showed that the mean HBL in the study cohort was 923.93 ± 489.04 mL, accounting for 78.76% ± 16.09% of the total perioperative blood loss, which averaged 1,123.39 ± 518.89 mL.

Univariate analysis identified hypertension as a significant risk factor for occult blood loss, and multiple linear regression confirmed hypertension as an independent predictor of hip fracture-related blood loss (HBL) (*p* < 0.05). These findings are consistent with previous research (24). The underlying pathophysiological mechanisms are thought to involve vascular endothelial dysfunction and altered hemostasis. Chronic hypertension has been shown to reduce tight junction proteins (e.g., ZO-1) by 30%–50%, impairing vascular barrier integrity and facilitating erythrocyte extravasation (25–27). Additionally, hypertensive patients exhibit elevated D-dimer levels (1.5- to 2.2-fold increase), suggesting enhanced fibrinolysis and impaired clot stability, a phenomenon also observed in atherosclerotic populations (28). Collectively, these molecular changes contribute to the exacerbation of microvascular leakage.

Notably, unlike the study by Foss et al., which focused solely on surgical factors (29), the present study explores the relationship between hypertension stratification and occult blood loss. Our findings demonstrated a significant increase in HBL in THA patients with hypertension ≥ grade 2 (i.e., systolic blood pressure ≥160 mmHg), with a dose-dependent effect (grade 2: 11.2% increase; grade 3: 18.7% increase). This result extends beyond previous analyses of surgical risk factors, suggesting the presence of a potential clinical threshold. When systolic blood pressure consistently exceeds 160 mmHg, the hemodynamic load increases significantly, exacerbating endothelial damage and potentially increasing the structural fragility of the microvasculature, making it more prone to rupture and bleeding during surgery. Literature has shown that hemodynamic instability, characterized by excessive blood pressure fluctuations during surgery, exacerbates HBL by increasing capillary shear stress and compromising endothelial glycocalyx integrity (30, 31). These findings highlight the critical importance of meticulous preoperative blood pressure management.

Although not statistically significant (*p* = 0.277), the interaction between hypertension and hypoalbuminemia resulted in an increase in HBL by 119.60 mL (11.30%). This non-significant result could be due to the relatively small sample size in the low albumin subgroup and the considerable inter-individual variability in blood loss. Nonetheless, its potential clinical relevance should not be overlooked. The study also found a significant correlation between blood loss and transfusion likelihood, with each additional unit of blood loss increasing the probability of requiring a transfusion. Similarly, Porcaro et al. reported that for every 1 mL increase in intraoperative blood loss, the risk ratio for blood transfusion was 1.002 (32, 33). These findings suggest that metabolic disorders may exacerbate vascular permeability through multiple mechanisms. Hypoalbuminemia, by reducing plasma colloid osmolality, creates a gradient that further worsens hypertension-induced endothelial dysfunction (34–36). Our result echoes the view proposed by Hosseini-Monfared et al. (37), reinforcing previous studies linking metabolic syndrome to increased surgical complications and emphasizing the need for preoperative metabolic optimization.

Based on the dose-response relationship observed between hypertension grading and HBL, we propose a stratified blood pressure management protocol that integrates preoperative, intraoperative, and metabolic management. This multifaceted approach is intended to enhance therapeutic outcomes through targeted interventions. Specifically, we recommend setting the preoperative systolic blood pressure target below 160 mmHg to intervene effectively in high-risk patients with grade 2 or higher hypertension. This strategy aims to reduce shear stress on the microvascular endothelial barrier caused by excessive hemodynamic pressure, thereby minimizing vascular leakage at its source. However, it is important to note that this threshold is based on retrospective data and should be validated in prospective studies. In addition, continuous intraoperative hemodynamic monitoring is essential to maintain blood pressure stability, preventing endothelial glycocalyx shedding and capillary stress injuries due to excessive blood pressure fluctuations, thereby mitigating intraoperative exacerbation of HBL. Furthermore, correcting metabolic disturbances such as hypoalbuminemia can increase plasma colloid osmotic pressure, reinforcing vascular wall integrity and synergistically enhancing blood pressure management to reduce erythrocyte extravasation. This comprehensive, multimodal management strategy is expected to reduce HBL by 11%–19% in high-risk patients, decrease transfusion requirements, and improve early rehabilitation outcomes by optimizing perioperative physiological conditions, ultimately enhancing postoperative recovery.

### Limitations

This study has several limitations. First, its retrospective, single-center design introduces the risk of selection bias and limits the external validity of our findings, making it difficult to generalize the results to other populations or clinical settings. Second, key perioperative data were neither standardized nor consistently available, which may have affected the robustness of our analysis. In particular, the lack of ambulatory blood pressure monitoring precluded the evaluation of blood pressure variability, a potential confounder that could have influenced the assessment of hidden blood loss. Furthermore, although certain surgical variables, such as operative approach, duration, and surgeon experience, were not included in the analysis, we also note the absence of standardized data on intraoperative use of hemostatic agents, a critical factor that may have influenced bleeding outcomes. Third, while hypertension was identified as an independent risk factor for blood loss, our analysis could not account for all relevant confounders. Specifically, we were unable to assess the differential effects of various classes of antihypertensive medications on bleeding risk due to the unavailability of detailed pharmacological data. Finally, while the estimation of hidden blood loss using the Gross formula is widely used and practical, it is based on theoretical calculations that rely on estimated blood volumes, which may not fully capture the complex dynamics of perioperative fluid shifts.

## Summary and outlook

In conclusion, this study confirmed hypertension as a risk factor for hidden blood loss (HBL) through a retrospective analysis of 1,035 cases. The stratified analysis demonstrated that HBL increased in a dose-dependent manner when hypertension reached ≥ grade 2. Additionally, metabolic disorders such as hypoalbuminemia further exacerbated HBL. Therefore, we recommend the implementation of individualized strategies for hypertensive patients, including preoperative control of systolic blood pressure to <160 mmHg and optimization of metabolic indices. These measures are expected to reduce HBL and subsequently lower the rate of postoperative transfusions in hypertensive patients undergoing total hip arthroplasty (THA).

## Data Availability

The original contributions presented in the study are included in the article/Supplementary Material, further inquiries can be directed to the corresponding author/s.

## References

[1] Kahn TL Frandsen JJ Blackburn BE Anderson LA Pelt CE Gililland JM Anterior-based approaches to total hip arthroplasty: beyond the learning curve. J Arthroplasty. (2022) 37(7):S552–5. 10.1016/j.arth.2022.01.042 35241320

[2] Bloch BV White JJE Matar HE Berber R Manktelow ARJ. Should patient age thresholds dictate fixation strategy in total hip arthroplasty? Bone Joint J. (2022) 104-B(2):206–11. 10.1302/0301-620X.104B2.BJJ-2021-1199.R1 35094580

[3] Jaramillo S Montane-Muntane M Gambus PL Capitan D Navarro-Ripoll R Blasi A. Perioperative blood loss: estimation of blood volume loss or haemoglobin mass loss? Blood Transfus. (2019) 18(1):20. 10.2450/2019.0204-19 31855150 PMC7053522

[4] Sun Y Miao H Gong H Zhang Y Hong W. Risk factors analysis and nomogram model establishment of hidden blood loss in overweight and obese elderly patients after total hip arthroplasty. Clin Interv Aging. (2024) 19:57–66. 10.2147/CIA.S428307 38223134 PMC10788052

[5] Zhu J Xu C Jiang Y Zhu J Tu M Yan X. Development and validation of a machine learning algorithm to predict the risk of blood transfusion after total hip replacement in patients with femoral neck fractures: a multicenter retrospective cohort study. Orthop Surg. (2024) 16(8):2066–80. 10.1111/os.14160 38951965 PMC11293940

[6] Li G Yu F Liu S Weng J Qi T Qin H Patient characteristics and procedural variables are associated with length of stay and hospital cost among unilateral primary total hip arthroplasty patients: a single-center retrospective cohort study. BMC Musculoskelet Disord. (2023) 24(1):6. 10.1186/s12891-022-06107-w 36600222 PMC9811718

[7] Lewis SR Macey R Parker MJ Cook JA Griffin XL. Arthroplasties for hip fracture in adults. Cochrane Database Syst Rev. (2022) 2(2):CD013410. 10.1002/14651858.CD013410.pub2 35156194 PMC8841979

[8] Liu J Lei Y Liao J Liang X Hu N Huang W. Pre-emptive antifibrinolysis: its role and efficacy in hip fracture patients undergoing total hip arthroplasty. J Arthroplasty. (2022) 37(4):755–62. 10.1016/j.arth.2021.12.034 34979252

[9] Li ZJ Zhao MW Zeng L. Additional dose of intravenous tranexamic acid after primary total knee arthroplasty further reduces hidden blood loss. Chin Med J. (2018) 131(06):638–42. 10.4103/0366-6999.226884 29521284 PMC5865307

[10] Gallo G Volpe M Savoia C. Endothelial dysfunction in hypertension: current concepts and clinical implications. Front Med (Lausanne). (2022) 8:798958. 10.3389/fmed.2021.798958 35127755 PMC8811286

[11] Bellien J Iacob M Richard V Wils J Cam-Duchez VL Joannides R. Evidence for wall shear stress-dependent t-PA release in human conduit arteries: role of endothelial factors and impact of high blood pressure. Hypertens Res. (2021) 44(3):310–7. 10.1038/s41440-020-00554-5 32943781

[12] Durante A Mazzapicchi A Baiardo Redaelli M. Systemic and cardiac microvascular dysfunction in hypertension. Int J Mol Sci. (2024) 25(24):13294. 10.3390/ijms252413294 39769057 PMC11677602

[13] Hong WS Zhang YX Lin Q Sun Y. Risk factors analysis and the establishment of nomogram prediction model of hidden blood loss after total hip arthroplasty for femoral neck fracture in elderly women. Clin Interv Aging. (2022) 17:707–15. 10.2147/CIA.S363682 35548382 PMC9081002

[14] Cui H Chen K Lv S Yuan C Wang Y. An analysis of perioperative hidden blood loss in femoral intertrochanteric fractures: bone density is an important inXuencing factor. BMC Musculoskelet Disord. (2021) 22(1):6. 10.1186/s12891-020-03922-x 33397328 PMC7784311

[15] Wei D Jiang Y Long X Huang N Xiang J Cheng J Predicting the perioperative transfusion risk of proximal femoral antirotation nailing (PFNA) for elderly patients with intertrochanteric fractures: a new predictive nomogram. Biomed Eng Online. (2025) 24(1):80. 10.1186/s12938-025-01419-z 40598529 PMC12220181

[16] Cap AP Beckett A Benov A Borgman M Chen J Corley JB Whole blood transfusion. Mil Med. (2018) 183(suppl_2):44–51. 10.1093/milmed/usy120 30189061

[17] Nadler SB Hidalgo JU Bloch T. Prediction of blood volume in normal human adults. Surgery. (1962) 51(2):224–32. 21936146

[18] Gross JB. Estimating allowable blood loss: corrected for dilution. Anesthesiology. (1983) 58(3):277–80. 10.1097/00000542-198303000-00016 6829965

[19] Carey RM Whelton PK. 2017 ACC/AHA hypertension guideline writing committee*. prevention, detection, evaluation, and management of high blood pressure in adults: synopsis of the 2017 American College of Cardiology/American Heart Association hypertension guideline. Ann Intern Med. (2018) 168(5):351–8. 10.7326/M17-3203 29357392

[20] Yoon PW Kim HJ Yoon JY Moon J Lee S. The efficacy of oxidised regenerated cellulose powder in reducing blood loss during primary total hip arthroplasty: a retrospective cohort study. J Orthop Surg Res. (2025) 20(1):368. 10.1186/s13018-025-05782-4 40211364 PMC11987413

[21] Jin JF Chen HR Peng YJ Dai J Wang QL Yan J. Risk factors for hidden blood loss in unilateral biportal endoscopic lumbar interbody fusion: a single-center retrospective study. BMC Musculoskelet Disord. (2024) 25(1):1–7. 10.1186/s12891-024-08104-7 39695594 PMC11657688

[22] Nehls F Schlppi M Madjdpour C Meier C Wahl P. Blood loss in primary total hip arthroplasty occurs mainly postoperatively, but current formulas for calculating blood loss are inaccurate: a retrospective study of 208 cases. Arch Orthop Trauma Surg. (2025) 145(1):283. 10.1007/s00402-025-05815-x 40343520

[23] Wood RC III Stewart DW Slusher L El-Bazouni H Cluck D Freshour J Retrospective evaluation of postoperative bleeding events in patients receiving rivaroxaban after undergoing total hip and total knee arthroplasty: comparison with clinical trial data. Pharmacotherapy. (2015) 35(7):663–9. 10.1002/phar.1608 26095331

[24] Wang T Guo J Hou Z. Risk factors for perioperative hidden blood loss after intertrochanteric fracture surgery in Chinese patients: a meta-analysis. Geriatr Orthop Surg Rehabil. (2022) 13:21514593221083816. 10.1177/21514593221083816 35295824 PMC8918975

[25] El Bakkouri Y Chidiac R Delisle C Corriveau J Cagnone G Gaonac’h-Lovejoy V ZO-1 interacts with YB-1 in endothelial cells to regulate stress granule formation during angiogenesis. Nat Commun. (2024) 15(1):4405. 10.1038/s41467-024-48852-7 38782923 PMC11116412

[26] Hansen ES Rinde FB Edvardsen MS Hindberg K Latysheva N Aukrust P Elevated plasma D-dimer levels are associated with risk of future incident venous thromboembolism. Thromb Res. (2021) 208:121–6. 10.1016/j.thromres.2021.10.020 34763296

[27] Chen YC Huang AL Kyaw TS Bobik A Peter K. Atherosclerotic plaque rupture: identifying the straw that breaks the camel’s back. Arterioscler Thromb Vasc Biol. (2016) 36(8):e63–72. 10.1161/ATVBAHA.116.307993 27466619

[28] Degroote C von Känel R Thomas L Zuccarella-Hackl C Messerli-bürgy N Saner H Lower diurnal HPA-axis activity in male hypertensive and coronary heart disease patients predicts future CHD risk. Front Endocrinol (Lausanne). (2023) 14:1080938. 10.3389/fendo.2023.1080938 36967749 PMC10036761

[29] Foss NB Kehlet H. Hidden blood loss after surgery for hip fracture. J Bone Joint Surg Br. (2006) 88(8):1053–9. 10.1302/0301-620X.88B8.17534 16877605

[30] Zhao N Liu C Tian X Yang J Wang T. Acute brain injury increases pulmonary capillary permeability via sympathetic activation-mediated high fluid shear stress and destruction of the endothelial glycocalyx layer. Exp Cell Res. (2024) 434(2):113873. 10.1016/j.yexcr.2023.113873 38092346

[31] Cheng Z Zhang L Liu M Liang D Huang X Peng L. Factors inXuencing preoperative blood pressure Xuctuations in patients undergoing elective surgery: a retrospective observational study. Int J Gen Med. (2025) 18:1615–22. 10.2147/IJGM.S507706 40130073 PMC11930843

[32] Newman JM Webb MR Klika AK Murray TG Barsoum WK Higuera CA. Quantifying blood loss and transfusion risk after primary vs conversion total hip arthroplasty. J Arthroplasty. (2017) 32(6):1902–9. 10.1016/j.arth.2017.01.038 28236548

[33] Porcaro AB Rizzetto R Amigoni N Tafuri A Shakir A Tiso L Severe intraoperative bleeding predicts the risk of perioperative blood transfusion after robot-assisted radical prostatectomy. J Robot Surg. (2022) 16(2):463–71. 10.1007/s11701-021-01262-z 34131882 PMC8960588

[34] Valeriani E Pannunzio A Palumbo IM Bartimoccia S Cammisotto V Castellani V Risk of venous thromboembolism and arterial events in patients with hypoalbuminemia: a comprehensive meta-analysis of more than 2 million patients. J Thromb Haemostasis. (2024) 22(10):2823–33. 10.1016/j.jtha.2024.06.018 38971499

[35] Wiedermann CJ. Moderator effect of hypoalbuminemia in volume resuscitation and plasma expansion with intravenous albumin solution. Int J Mol Sci. (2022) 23(22):14175. 10.3390/ijms232214175 36430652 PMC9695189

[36] McLean C Mocanu V Birch DW Karmali S Switzer NJ. Hypoalbuminemia predicts serious complications following elective bariatric surgery. Obes Surg. (2021) 31(10):4519–27. 10.1007/s11695-021-05641-1 34378157

[37] Hosseini-Monfared P Mirahmadi A Sarzaeem MM Pourshahryari S Aminnia P Poursalehian M Ascorbic acid reduces the blood boss after total knee arthroplasty: insights from a randomized controlled trial. Arthroplasty Today. (2025) 32:101618. 10.1016/j.artd.2025.101618 39974338 PMC11836488

